# Inequities in access to healthcare: analysis of national survey data across six Asia-Pacific countries

**DOI:** 10.1186/1472-6963-13-238

**Published:** 2013-07-01

**Authors:** Samantha B Meyer, Tini CN Luong, Loreen Mamerow, Paul R Ward

**Affiliations:** 1 Discipline of Public Health, Flinders University, Sturt Road, Bedford Park, South Australia 5042, Australia

**Keywords:** Equity, Access, Healthcare, Asia, Pacific, Social determinants, Policy

## Abstract

**Background:**

Evidence suggests that there is a link between inequitable access to healthcare and inequitable distribution of illness. A recent World Health Organization report stated that there is a need for research and policy to address the critical role of health services in reducing inequities and preventing future inequities. The aim of this manuscript is to highlight disparities and differences in terms of the factors that distinguish between poor and good access to healthcare across six Asia-Pacific countries: Australia, Hong Kong, Japan, South Korea, Taiwan, and Thailand.

**Methods:**

A population survey was undertaken in each country. This paper is a secondary analysis of these existing data. Data were collected in each country between 2009 and 2010. Four variables related to difficulties in access to healthcare (distance, appointment, waiting time, and cost) were analysed using binomial logistic regression to identify socio- and demographic predictors of inequity.

**Results:**

Consistent across the findings, poor health and low income were identified as difficulties in access. Country specific indicators were also identified. For Thailand, the poorest level of access appears to be for respondents who work within the household whereas in Taiwan, part-time work is associated with difficulties in access. Within Hong Kong, results suggest that older (above 60) and retired individuals have the poorest access and within Australia, females and married individuals are the worst off.

**Conclusion:**

Recognition of these inequities, from a policy perspective, is essential for health sector policy decision-making. Despite the differences in political and economic climate in the countries under analysis, our findings highlight patterns of inequity which require policy responses. Our data should be used as a means of deciding the most appropriate policy response for each country which includes, rather than excludes, socially marginalised population groups. These findings should be of interest to those involved in health policy, but also in policy more generally because as we have identified, access to health care is influenced by determinants outside of the health system.

## Introduction

Both trans-nationally [1-3] and within particular countries [4], it is widely recognised that public health policy and practice needs to focus on addressing the Social Determinants of Health (SDH) in order to increase the health of the most vulnerable and disadvantaged groups [5]. The focus on promoting health and preventing illness for the most vulnerable groups in society has been part of the named agenda of public health for decades, and is central to contemporary global agreements such as the Jakarta Declaration [6] and the Bangkok Charter for Health Promotion [7]. Indeed, Professor Sir Michael Marmot recently referred to health inequities as a “stain on our society” which requires concerted political will and moral imperative to change [8].

Equitable access to healthcare services is a major SDH [1,3], with a WHO report stating there is a need for research and policy to address the critical role of “health services in reducing ill health and suffering, redressing inequities, and preventing future inequities” [9]. The WHO Commission on the SDH provides evidence on the link between inequitable access to healthcare and the resulting inequitable distribution of illness and make a recommendation for interventions to increase the equity of access to healthcare services [10-12].

There is a large research literature demonstrating that access to, quality of, and outcomes from healthcare are inequitable across a number of clinical areas including screening for a variety of cancers [13-16], surgical interventions [17-19] and primary care prescribing [20-22]. These examples provide evidence for the ‘inverse care law’ [23], whereby the groups with the greatest healthcare need receive the lowest levels of service. This evidence has led to an international call for action to reduce inequities in health and healthcare [1,24,25].

The incorporation of equity in health provision helps to identify the significant roles of political and social consciousness in developing and sustaining the needs and agendas of all people in a fair and just way [26]. For the purposes of this manuscript, a key distinction needs to be made between a relatively large field of research which defines equity in health [27-30] and an emerging field of research which defines equity in healthcare [31-34]. It is hypothesised that improving equity in access to healthcare will lead to improved equity in health outcomes [11].

This manuscript presents for the first time, a secondary analysis of cross-country data which identifies populations at risk of poor access to healthcare in six Asia-Pacific countries: Australia, Hong Kong, Japan, South Korea, Taiwan, and Thailand. Specifically, this manuscript reports the findings of a population survey undertaken in each country regarding difficulties in access to healthcare: namely, difficulties due to distance, obtaining an appointment, waiting times and costs. The aim of this manuscript is to highlight disparities and differences in terms of the factors that seem to be important to distinguishing between poor and good access to healthcare within each country.

## Background

Healthcare is socially determined in that it is influenced by social policies that govern the receipt and utilization of health services, allocation of healthcare resources, the financing of healthcare, and the quality of healthcare services [35]. Ensuring that appropriate resources are mobilized to meet the healthcare needs of different groups in different populations is key to healthcare access [36]. The notion of ‘equity’ is inextricably linked to the concept of access to healthcare, because the inclusion of fairness or social justice is one of the key factors necessary for the effective provision of healthcare [37].

There is a great deal of research focused on defining equity in access to healthcare [36,38-40], and its operationalisation [41-44]. The literature on “equity” and “access” to healthcare is abundant, diverse, and complex and the conceptualisation and operationalisation of these two concepts is not consistent [44]. Many studies have typically relied on measures of utilization as the basis for analyses of equity of access, although this is methodologically and conceptually problematic because it does not measure need and thus, does not identify problems of inequity. This study diverts the focus away from measuring utilization of health services and instead, measures socio-demographics against healthcare access indicators. We then discuss these findings in relation to healthcare provision within each of these six countries.

We conceptualise access as the level of facilitation or inhibition affecting the ability of individuals to gain entry to and receive care from healthcare services [45]. The accessibility of healthcare depends on a multitude of supply- and demand-side factors [46]. Supply-side factors include the distribution of facilities, waiting times, and human resources and capital [36,47,48]. Factors influencing accessibility on the demand-side result from predisposing, enabling, and needs factors [49], including socio-demographics, past experiences with healthcare, perceptions regarding health and illness, income levels and scope, and extent of health insurance coverage [50]. Our conceptualisation of access was used to guide our investigation which looks specifically at the supply-side factors affecting access (cost, waiting time, appointment, distance) and relevant demand-side factors (socio-demographic and health related predictors).

An equitable health service implies that individuals’ utilisation of and access to service depends on their health state alone, and not upon their socio-economic status [51]. The research presented herein is based on our argument that this notion should be extended and access should not be based upon age, race, geography, gender, language, culture, and functional capacity. Equity in healthcare should be concerned with the reduction of systematic discrimination and marginalization between different groups and within groups, regardless of whether a group is found to attain a generally high or low level of equitable access. Our use of socio-demographic variables (e.g. sex, age, income) and variables related to the beliefs and expectations about health and illness (e.g. chronic health condition, self-rated health) help to reveal the differing levels and standards of needs, and have been included for analysis in this study.

## Methods

The data presented in this manuscript come from a larger survey designed to investigate social quality [52] across six Asia-Pacific counties: Australia, Hong Kong, Japan, South Korea, Taiwan, and Thailand. The survey was developed, and data collected, by academic representatives from each of the universities involved; Australia (Flinders University), Hong Kong (Chinese University Hong Kong), Japan (Chiba University), Korea (Seoul National University), Taiwan (National Taiwan University), Thailand (King Prajadhipok Institute). The survey was then translated into the language of the host country and validated. The survey was then piloted in each language and tested for reliability (see [53] for an overview of the validity and reliability testing in Australia). All questions asked were the same, but in a different language. The authors undertook data collection in Australia only and therefore, this paper reports a secondary analysis of a combined dataset representing all countries involved. The merging and cleaning of the dataset was conducted by academics at Seoul National University. Details of method for selected countries represented in this paper are published elsewhere [54-57]. Data were collected in each country between 2009 and 2010.

Four variables related to difficulties in access to healthcare (distance, appointment, waiting time, and cost) were selected and form the basis of this manuscript. The survey question regarding access is provided in Table 1.

**Table 1:** Survey question ‘On the last occasion you needed to see a doctor or medical specialist, to what extent did each of the following factors make it difficult for you to do so?’

	**Very**	**A little**	**Not**	**Not applicable / never**
	**difficult**	**difficult**	**difficult**	**needed to see doctor**
			**at all**	
Distance to doctor’s office /	□	□	□	□
hospital / medical centre				
Delay in getting appointment	□	□	□	□
Waiting time to see doctor on	□	□	□	□
day of appointment				
Cost of seeing the doctor	□	□	□	□

Ten independent variables (sex, age, marital status, work status, income, financial situation in the last year, subjective health satisfaction, self-rated health, perception of importance of health, and chronic health condition) were tested against the four dependent variables based on previous studies linking their relevance to equity of access to healthcare [47,58,59].

Data were analysed using the SPSS version 17.0 (SPSS Inc., Chicago, IL, USA). Binomial logistic regression models were used to investigate associations for all six countries [60]. In order to conduct binomial regression, the original four dependent variables were recoded into new variables with two categories of response. ‘Very difficult’ and ‘a little difficult’ were recoded as ‘difficult’. Responses ‘not applicable/never needed to see a doctor’ were coded as missing. ‘Not difficult at all’ was not recoded. Due to differences in data collection methods, and changes to survey questions to make them culturally relevant, a few of the independent variables were not available from some countries thus reducing the number of association tests performed. Goodness-of-fit for all models were checked [60,61].

To ensure that there would be no redundant calculations during the multivariate analyses, co linearity diagnostics (using SPSS) were performed to check for variables that may have similar degrees of variance [62]. A tolerance value of ≤ 0.20 and a variance inflation factor of ≥10 were used to indicate a multicollinearity problem [63]. Diagnostics were conducted for all 24 models (six countries by four dependent variables).

Some methodological issues were faced when the data from all six countries were checked for consistency. Rice et al. (2010:82) identify that the design of International surveys needs to be mindful of the requirement for the cross-cultural equivalence of instruments [64]. In order to address this, we consulted with academics who comprise the Asia-Pacific Scientific Steering Group on Social Quality (PR Ward is a founding member of this group) [2]. It was decided among committee members that options provided in the Australian survey were ‘very difficult’, ‘difficult’, or ‘not difficult at all’. Based on the recommendations of the committee member in Asia, response options were: ‘very difficult’, ‘difficult’, ‘easy’, and ‘very easy’. Additionally, it was decided that the wording for one independent variable, subjective health satisfaction, differed between the Australian survey and all other countries’ surveys; Australia’s item options were ‘happy’, ‘average’, and ‘unhappy’ and all other countries’ were ‘satisfied’, ‘neither satisfied nor dissatisfied’, and ‘unsatisfied’.

Robone et al. (2011) also identify difficulties in using scale variables in cross-country comparisons because there is potential that when faced with the instrument individuals are likely to interpret the meaning of the available response categories in a way that systematically differs across populations or population subgroups [65] which compromises the comparability of the data in cross-country analyses. We recognise that by applying country level estimates within the analysis (mixed effects logistic regression models [66]), we can draw more insightful conclusions for comparing the data. However, this form of analysis in not in line with the aim of this manuscript which is to report on within country results rather than across country comparisons.

Table 2 summarizes the methods employed for the collection of data within Australia, Japan, South Korea, Hong Kong, Taiwan (Taipei), and Thailand. It outlines the methodological issues which are discussed in more detail as limitations in the conclusion of the paper.

**Table 2:** Comparison of survey methods for six countries

	**Australia**	**Japan**	**South Korea**	**Hong Kong**	**Taiwan-Taipei**	**Thailand**
**Fieldwork dates**	Sept. 2009	11 Sept. ~ 21 Sept.	20 Oct. ~ 10	October 2009 ~	6 Oct. ~ 16	20 Oct. ~ 10 Nov.,
		2009	Nov., 2009	October 2010	Nov., 2009	2009
**Population**	18 years old and	19 years old and	18 years old and	18 years old or	Taipei legal	18 years old and
	over	over	over	above	citizens who	over
					are 20 years	
					old or older	
**Geographic**	Nationwide	Nationwide	Nationwide	Hong Kong	Taipei	Nationwide
**coverage**						
**Sampling method**	Stratified according	Regionally stratified	Multi – Stages	Quota sampling	Central	Multi – Stages
	to state population	proportional	Sampling	based on the	Location Quota	Sampling
		random sampling		three age ranges	Sampling	
**Fieldwork**	Self-administered	Face to face	Face to face	Telephone	Face to face	Face to face
**Methods**	postal survey	interview	interview	interview	interview	interview
**Sample size**	1,044	1,006	1,200	681	1,200	1,200

Appropriate approvals were obtained within each country to undertake the individual surveys. The authors were granted ethics approval from Flinders University Social and Behavioural Research Ethics Committee to obtain and use the collected data for secondary analysis (project number 5221).

## Results

The reports below are inclusive of only statistics regarding the predictor variables. Table 3 summarizes all of the predictors found to be significant for each of the four dependent variables.

**Table 3:** Summary of variables associated with difficulties in access to healthcare

**Country**		**Difficulties due to distance**	**Difficulties in getting**	**Difficulty due to**	**Difficulties due to cost**
			**an appointment**	**waiting times**	
Australia	Demographic	Married, divorced, widowed or	Female; married	Female; worked part-	Female; married or
		separated		time	cohabiting
	Health	Felt very unhappy with their health;	Nil	Nil	Nil
	related				
	Economic	S some savings and borrowed	Just got by, spent	Just got by financially	Second-lowest income
		money; or fell in the lowest income	savings, or spent	in the last year, spent	quartile; just got by
		quartile group	savings and borrowed	some savings or spent	financially or spend
			money	some savings *and*	savings *and* borrowed
				borrowed money	money
Hong Kong	Demographic	Nil	Aged 60 years or older	Retired or pensioner	Retired/pensioner
	Health	Nil	Nil	Nil	Perceived health to be
	related				neither important nor
					unimportant
	Economic	Spent some savings or fell in the lowest	Nil	Nil	Nil
		income quartile group			
Japan	Demographic	Nil	Nil	30–39 years of age	Nil
	Health	Rated their health as very bad	Satisfied with their	Rated their health as	Rated their health as fair, or
	related		health	good, fair, bad, or	rated their health as bad
				very bad	
	Economic	Nil	Nil	Nil	Spent some savings or spent some savings and borrowed money
Korea	Demographic	Nil	Nil	Nil	Female
	Health	Fair state of health, very bad health;	Nil	Nil	Nil
	related	chronic health condition			
	Economic	Nil	Nil	Nil	Spent savings and borrow
					money
Taiwan	Demographic	Divorced; part-time workers	Nil	Nil	Work part-time
	Health	Nil	Nil	Nil	Nil
	related				
	Economic	Spent their savings or spent their	Nil	Spend some savings	Just got by, spent savings,
		savings and borrowed			spent savings *and*
					borrowed money
Thailand		Work for their family, a	Work for their family,	Work for family	Nil
		housewife/househusband or a	work part-time		
		student/unemployed/other			
		Rated their health as being good, fair or	Nil	Nil	Nil
		bad			
		Just got by financially, spent some	Nil	Just got by	Spend savings, or spend
		savings or spent some savings *and*		financially, or spent	savings *and* borrowed
		borrowed money; and		their savings *and*	money
				borrowed money	

Statistically significant outputs for all binomial regression models for all predictor variables are presented below. Within the following section, any odds ratios of less than one should be interpreted as relating to poor access for the particular group within which we are referring.

### Distance

Table 4 provides statistical results from binomial logistic regression models computed for the dependent variable distance.

**Table 4:** Six binomial logistic regression models for dependent variable: distance

**Country**	**1. AU**	**2. HK**	**3. JP**	**4. KR**	**5. TW**	**6. TL**
**(Models for DV1)**						
Model fit χ^2^ (df)	110.68 (15)**	55.69(6)***	52.00(8)***	39.94(5)***	34.96(14)**	78.43(13)***
N response/N total	1029/1044	641/674 (95.1%)	996/1000 (99.6%)	955/1006 (94.9%)	1089/1200 (90.8%)	1185/1200 (98.8%)
Nagelkerke R^2^	0.28	0.16	0.08	0.07	0.05	0.10
*Independent Variable*	*Waldχ*^*2*^(df) p*	*Waldχ*^*2*^(df) p*	*Waldχ*^*2*^df) p*	*Waldχ*^*2*^(df) p*	*Waldχ*^*2*^(df) p*	*Waldχ*^*2*^(df) p*
	*OR (95%CI)*	*OR (95%CI)*	*OR (95%CI)*	*OR (95%CI)*	*OR (95%CI)*	*OR (95%CI)*
	*Coefficient b*	*Coefficient b*	*Coefficient b*	*Coefficient*	*Coefficient b*	*Coefficient b*
**Marital status**	21.35 (5)**	”	’	’	11.17χ^2^(5)*	
Never married (ref)	OR 1.00				OR 1.00	
	0				0	
Married	14.69χ^2^ (1)***	’	’	’	’	’
	0.26 (0.13–0.52)					
	−1.34					
Divorced	‘	’	’	’	8.83χ^2^(1)**	’
					0.34 (0.17–0.69)	
					−1.07	
Widowed	11.46χ^2^ (1)**	’	’	’		’
	0.21 (0.08–0.52)					
	−1.57					
Separated	6.75χ^2^ (1)**	’	’	’		’
	0.22 (0.07–0.70)					
	−1.50					
Cohabiting		’	’	’		’
**Work status**	’	’	’	’	13.73χ^2^(6)*	24.24χ^2^(6)***
					1.00	1.00
Full-time (ref)					0	0
Part-time	’	’	’	’	5.76χ^2^(1)*	‘
					0.50 (0.28–0.88)	
					−0.70	
Self-employed	’	’	’	’	‘	.
Work for family	’	’	’	’	‘	12.92χ^2^(1)***
						0.47 (0.31–0.71)
						−0.76
Retired/	’	’	’	’	4.56χ^2^(1)*	‘
Pensioner					2.00 (1.06–3.77)	
					0.69	
Housewife/	’	’	’	’	‘	10.24χ^2^(1)**
Househusband						0.42 (0.25–0.72)
						−0.86
Student/	’	’	’	’	‘	6.42χ^2^(1)*
Unemployed/						0.59 (0.40–0.89)
Other						−0.54
**Subjective health**	24.67χ^2^ (2)***	*’*	***Dissatisfied (ref)***	*’*	*’*	***Dissatisfied (ref)***
**Satisfaction**	1.00		12.50χ^2^(2)**			7.54χ^2^(2)*
Unhappy (ref)	0		1.00			1.00
			0			0
Average	14.36χ^2^ (1)***	*’*	***Average***	*’*	*’*	***’***
	3.15 (1.74–5.69)		12.34χ^2^(1)***			
	1.15		2.18 (1.41–3.37)			
			0.54			
Happy	24.52χ^2^ (1)***	***’***	***Satisfied***	***’***	***’***	***Satisfied***
	3.91 (2.28–6.71)		5.32χ^2^(1)*			6.35χ^2^(1)*
	1.36		1.72 (1.09–2.72)			1.85 (1.15–2.98)
			0.54			0.61
**Financial situation in**	12.88χ^2^ (3)**	21.52χ^2^(3)***	***’***	***’***	8.71χ^2^(3)*	18.71χ^2^(3)***
**last year**	1.00	1.00			1.00	1.00
Save money (ref)	0	0			0	0
Just get by	‘	***’***	’	***’***	***’***	15.38χ^2^(1)***
						0.49 (0.35–0.70)
						−0.71
Spent some savings	’	11.11χ^2^(1)**	’	’	5.82χ^2^(1)*	7.72χ^2^(1)**
		0.37 (0.20–0.66)			0.51 (0.29–0.88)	0.47 (0.27–0.82)
		−1.00			−0.68	−0.70
Spent savings &	11.87χ^2^ (1)**	’	’	’	5.16χ^2^(1)*	13.80χ^2^(1)***
borrowed money	0.35 (0.19–0.63)				0.49 (0.26–0.91)	0.43 (0.27–0.67)
	−1.06				−0.72	−0.85
**Household annual or**	(annual)	(monthly)	’	’	’	’
**monthly adjusted**	9.04χ^2^ (3)*	16.18χ^2^(3)**				
**income quartile**	1.00	1.00				
4^th^: quartile (ref)	0	0				
1^st^ quartile	3.83χ^2^ (1)*	14.99χ^2^(1)***	’	’	’	’
	0.55 (0.30–1.02)	0.27 (0.14–0.52)				
	−0.59	−1.32				
2^nd^ quartile	5.31χ^2^ (1)*	’	’	’	’	’
	0.54 (0.32–0.93)					
	−0.62					
3^rd^: quartile	’	’	’	’	’	’
**Self-rated health**	’	’	14.76χ^2^(4)**	22.13χ^2^(4)***	’	11.47χ^2^(4)*
Very good (ref)			1.00	1.00		1.00
			0	0		0
Good	’	’	’	’		8.16χ^2^(1)**
						0.54 (0.35–0.82)
						−0.63
Fair	’	’	’	13.41χ^2^(1)***	’	10.29χ^2^(1)**
				0.43 (0.28–0.68_		0.47 (0.30–0.75)
				−0.84		−0.76
Bad	’	’	8.31χ^2^(1)**	’	’	4.99χ^2^(1)*
			0.31 (0.14–0.86)			0.44 (0.21–0.90)
			−1.19			−0.83
Very bad	’	’	’	6.79χ^2^(1)**	’	’
				0.25 (0.09–0.71)		
				−1.38		
**Whether respondent has**	’	’	’	–	’	’
**chronic health**				1.00		
**condition**				0		
Yes (ref)						
No	’	’	’	6.99(1)**	’	’
				3.34 (1.37–8.18)		
				1.21		

Ref: reference level, * p<.05; ** p<.01, *** p<.001.

#### Monthly/annual income

In Australia, respondents who fell in the highest annual income quartile were 45% more likely that those in the lowest income quartile [OR=0.55, CI=0.30–1.00, p<0.05] and second-lowest income quartile [OR=0.54, CI=0.32–0.91, p<0.05] to state that distance was not a difficulty factor.

In Hong Kong, respondents who fell in the highest annual income quartile were about 75% more likely to state that distance was not a difficulty factor than those from the lowest income quartile [OR=0.27, CI=0.14–0.52, p<0.001].

#### Subjective health satisfaction

In Australia, respondents who felt indifferent [OR=3.15, CI=1.74–5.69, p<0.001] or happy [OR=3.91, CI=2.28–6.71, p<0.001] about the state of their health were three or four times (respectively) more likely to indicate that distance was not a difficulty factor than those who stated they were very unhappy with their health.

Those who stated average satisfaction with their health in Japan [OR=2.18, CI=1.41–3.37, p<0.001] or said they were satisfied [OR=1.72, CI=1.09–2.72, p<0.05] were approximately twice as likely to state that distance was not difficult than those who indicated they were dissatisfied with their health.

In Thailand, respondents who stated they were satisfied with their health were about 1.9 times more likely [OR=1.85, CI=1.15–2.98, p<0.05] to state that distance was not a difficulty than those who stated they were dissatisfied with their health.

#### Financial situation

In Australia, respondents who stated they had saved money were about 65% more likely to state that distance was not a difficulty factor than those who stated they had spent savings and borrowed money [OR=0.35, CI=0.19–0.63, p<0.01].

Hong Kong respondents who stated they had saved money in the last year were about 65% more likely to state that distance was not a difficulty factor than those who stated they had spent some savings [OR=0.37, CI=0.20–0.66, p<0.01].

In Taiwan, respondents who stated they had saved money in the last year were about 50% more likely to state that distance was not a difficulty factor than those respondents who stated they had spent some savings [OR=0.51, CI=0.29–0.88, p<0.05] and respondents who stated they had spent savings and borrowed money [OR=0.49, CI=0.26–0.91, p<0.05].

Compared with respondents who stated they had spent some savings in Thailand [OR=0.47, CI=0.27–0.82, p<0.01] and respondents who selected the item ‘just got by’ [OR=0.49, CI=0.35–0.70, p<0.001], those who stated they had saved money in the last year in Thailand were about 50% more like to respond that distance was not a difficulty factor. Additionally, those who stated they had saved money in the last year were about 60% more likely than those who stated they had spent savings and borrowed money [OR=0.43, CI=0.27–0.67, p<0.001] to state that distance was not a difficulty factor.

#### Self-rated health (Not applicable to Hong Kong & Taiwan)

In Japan, respondents who rated their health as being very good were approximately 70% more likely to state that distance was not a difficulty factor than respondents who rated their health condition as bad [OR=0.31, CI=0.14–0.68, p<0.01].

Korean respondents who rated their health as very good were about 60% more likely to state ‘not difficult’ than those who felt that their health condition was ‘fair’ [OR=0.43, CI=0.28–0.68, p<0.001]. Those who rated their health as being very good were about 75% more likely to state that distance was not a difficulty factor than those who rated their health as being very bad [OR=0.25, CI=0.09–0.71, p<0.01].

In Thailand, those who rated their health as being very good were approximately 50% more likely to state that distance was not a difficulty factor compared with respondents who rated their health condition as good, fair, or bad [OR=0.54, CI=0.35–0.82, p<0.01; OR=0.47, CI=0.30–0.75, p<0.01; and OR=0.44, CI=0.21–0.90, p<0.01, respectively].

#### Chronic health condition (Not applicable to Hong Kong, Japan & Taiwan)

In Korea respondents who had no chronic health condition were nearly 3.5 times more likely [OR=3.34, CI=1.37–8.18, p<0.01] to state not difficult for distance than those who stated they had a chronic health condition.

#### Marital status

In Australia, those who stated they had never married were around 70–80% more likely to report distance was not a difficulty factor in seeing a doctor or a specialist on the last occasion they required compared with those who stated they were married [OR=0.26, CI=0.13–0.52, p<0.001], divorced [OR=0.19, CI=0.09–0.45, p<0.001] widowed [OR=0.21, CI=0.08–0.52, p<0.01] or separated [OR=0.22, CI=0.07–0.70, p<0.01].

In Taiwan, those who had never married were about 70% more likely than those who stated they were divorced [OR=0.34, CI=0.17–0.69, p<0.01] to state that it was not difficult in terms of distance to travel on the last occasion they required to see a doctor/specialist.

#### Work status

In Taiwan, respondents who worked full-time were approximately 50% more likely to state ‘not difficult’ in terms of distance than those who worked part-time [OR=0.50, CI=0.28–0.88, p<0.05]. Those who were retired/pensioners [OR=2.00, CI=1.06–3.77, p<0.05] were twice as likely as those who worked full-time to state that distance was not a difficulty factor the last time they required to see a doctor or specialist.

In Thailand, respondents who worked full-time were about 55% more likely to state that distance was not a difficulty factor than those who indicated that they worked for their family [OR=0.47, CI=0.31–0.71, p<0.001]. Respondents who worked full-time were found to be nearly 60% more likely than respondents who were housewives/househusbands were [OR=0.42, CI=0.25–0.72, p<0.01] and about 40% more likely than those who indicated student/unemployed/other [OR=0.59, CI=0.40–0.89, p<0.05] to find distance not a difficulty.

### Appointment

Table 5 provides statistical results for binomial logistic regression models computed for the dependent variable appointment.

**Table 5:** Six binomial logistic regression models for dependent variable: appointment

**Country**	**1. AU**	**2. HK**	**3. JP**	**4. KR**	**5. TW**	**6. TL**
**(Models for DV2)**						
Model fit χ^2^ (df)	125.79 (18)***	103.79(5)***	36.87(8)***	18.45(4)**	16.50(2)***	47.09(11)***
N response/N total	816/1044 (78.2%)	667/674 (99.0%)	996/1000 (99.6%)	990/1006 (98.4%)	1157/1200 (96.4%)	1185/1200 (98.8%)
Nagelkerke R^2^	0.19	0.19	0.06	0.03	0.02	0.05
*Independent Variable*	Waldχ^2^(df) p*	Waldχ^2^(df) p*	Waldχ^2^(df) p*	Waldχ^2^ df) p*	Waldχ^2^ (df) p*	Waldχ^2^(df) p*
	OR (95%CI)	OR (95%CI)	OR (95%CI)	OR (95%CI)	OR (95%CI)	OR (95%CI)
	Coefficient	Coefficient	Coefficient	Coefficient	Coefficient	Coefficient
**Sex**	-	’	’	’	’	’
Male (ref)	1.00					
	0					
Female	18.59χ^2^ (1)***	’	’	’	’	’
	00.50 (0.37–0.69)					
	−0.69					
**Age**	27.55χ^2^(5)***	82.60χ^2^(5)***	’	’	’	15.27(5)**
< 20 (ref)	1.00	1.00				1.00
	0	0				0
20–29 (ref)	20.94χ^2^(1)***	’	17.45χ^2^(4)**	’	’	’
	18.87 (3.69–31.65)		1.00			
	2.38		0			
30–39	12.02χ^2^(1)**	’	’	’	’	’
	6.97 (2.33–20.88)					
	1.94					
40–49	14.59χ^2^(1)***	’	’	’	’	’
	7.91 (2.62–23.91)					
	2.07					
50–59	16.74χ^2^(1)***	’	’	’	’	’
	10.43 (3.39–32.07)					
	2.35					
60+	20.83χ^2^(1)***	30.04χ^2^(1)***	11.62χ^2^(1)**	’	’	’
	13.86 (4.48–42.88)	0.13 (0.06–0.27)	2.37 (1.44–3.90)			
	2.63	−2.04	0.86			
**Marital status**	15.05χ^2^(5)**	’	’	’	’	’
Never married (ref)	1.00					
	0					
Married	12.56χ^2^(1)**	’	’	’	’	
	0.62 (0.27–0.70)					
	−0.83					
Divorced	’	’	’	’	’	
Widowed	’	’	’	’	’	
Separate	’	’	’	’	’	
Cohabiting	’	’	’	’	’	
Work status	’	’	’	’	’	34.93χ^2^(6)***
Full-time						1.00
						0
Part-time	’	’	’	’	’	6.37χ^2^(1)*
						0.45 (0.24–0.84)
						−0.80
Self-employed	’	’	’	’	’	’
Work for family	’	’	’	’	’	31.24χ^2^(1)***
						0.34 (0.23–0.49)
						−1.09
Retired/pensioner	’	’	’	’	’	’
Housewife/househusband	’	’	’	’	’	’
Student/	’	’	’	’	’	’
Unemployed/						
Other						
**Subjective health**	19.14χ^2^(2)***	’	’	***Dissatisfied (ref)***	***Dissatisfied (ref)***	’
**satisfaction**	1.00			15.90χ^2^(2)***	16.73χ^2^(2)***	
Unhappy (ref)	0			1.00	1.00	
				0	0	
Average	3.34χ^2^(1)	’	’	’	’	’
	1.78 (0.96–3.29)					
	0.57					
Happy	14.36χ^2^(1)***	’	’	***Satisfied***	***Satisfied***	’
	3.10 (1.73–5.56)			7.02χ^2^(1)**	15.20χ^2^(1)***	
	1.13			1.77 (1.16–2.71)	2.31 (1.52–3.51)	
				0.57	0.84	
**Financial situation in**	12.38χ^2^(3)**	’	’	’	’	’
**last year**	1.00					
Save money (ref)	0					
Just get by	9.85χ^2^(1)**	’	’	’	’	’
	0.56 (0.39–0.80)					
	−0.59					
Spent some savings	4.97χ^2^(1)*	’	’	’	’	’
	0.62 (0.40–0.94)					
	−0.49					
Spent savings &	5.70χ^2^(1)*	’	’	’	’	’
borrowed money	0.50 (0.28–0.88)					
	−0.69					
**Self-rated health**	’	’	26.46χ^2^(4)***	’	’	’
Very dissatisfied			1.00			
			0			
Dissatisfied	’	’	’	’	’	’
Average	’	’	’	’	’	’
Satisfied	’	’	10.30χ^2^(1)**	’	’	’
			0.31 (0.15–0.64)			
			−1.16			
Very satisfied	’	’	’	’	’	’
**Chronic health**	’	’	’	’	’	’
**condition**						
(Yes ref)						
No	3.83χ^2^(1)*	’	’	’	’	’
	1.41 (1.00–1.98)					
	0.34					

Ref: reference level, * p<.05; ** p<.01, *** p<.001.

#### Subjective health satisfaction

Australian respondents who stated they were happy about their health were about 3 times more likely than those who were unhappy about their health [OR=3.10, CI=1.73–5.56, p<0.001] and almost 2 times more likely than those who stated that there we ‘average’ about their health [OR=1.78, CI=0.96-3.29] to state that appointment delays were not a difficulty factor.

In Korea, those who stated they were satisfied with their health were nearly twice as likely [OR=1.77, CI=1.16–2.71, p<0.01] to state that appointment delay was not a difficulty factor compared with those who stated they were dissatisfied with their health.

In Taiwan respondents who stated they were satisfied with their health were nearly 2.5 times more likely [OR=2.31, CI=1.52–3.51, p<0.001] to state that appointment delay was not a difficulty factor than those who stated they were dissatisfied with their health.

#### Financial situation in last year

In Australia those who stated they had saved money were about 45% more likely to state that appointment delay was not a difficulty compared with those who ‘just got by’ [OR=0.56, CI=0.39–0.80, p<0.01]. Similarly, respondents who stated they had saved money were about 40% more likely to state that appointment delay was not a difficulty than those who had ‘spent some savings’ [OR=0.62, CI=0.40–0.94, p<0.05], and about 50% more likely to state that appointment delay was not a difficulty than those who had ‘spent savings and borrowed money’ were [OR=0.50, CI=0.28– 0.88, p<0.05].

#### Self-rated health

In Japan, those who stated they were very dissatisfied with their health were approximately 70% more likely to state that appointment delay was not a difficulty factor than those who stated they were satisfied with their health [OR=0.31, CI=0.15–0.64. p<0.01].

#### Chronic health condition and appointment difficulties

In Australia respondents who indicated they had no chronic health condition were nearly 1.5 times more likely [OR=1.41, CI=1.00–1.98, p<0.05] to state that appointment delays were not a difficulty than those who indicated having a chronic health condition.

#### Sex

Within Australia, males were about 50% more likely to state that it was not difficult to make an appointment the last time they required to see a doctor/GP than females [OR=0.50, CI=0.37–0.69, p<0.001].

#### Age

Australian respondents in all age groups from 20 years of age and over were at least 6 times more likely to state that making an appointment was not a difficulty factor. For example, those who were aged between 30–39 [OR=6.97, CI=2.33–20.88, p<0.001] were about 7 times more likely to state that appointment was not a difficulty factor, and those who were aged 60 or above were about 14 times more likely to state that appointment making was not a difficulty factor [OR=13.86, CI=4.48–42.89, p<0.001].

Age was the only significant predictor of appointment difficulties for Hong Kong. Respondents aged 20 years or younger were about 85% more likely to indicate that scheduling an appointment was not a difficulty factor on the last occasion they required to see a doctor/specialist than those who were aged 60 years or over [OR=0.13, CI=0.06–0.27, p<0.001].

In Japan, those who were 60 years or older were nearly 2.5 times more likely [OR=2.37, CI=1.44=3.90, p<0.01] to state that appointment making was not a difficulty factor on the last occasion they required to see a doctor/specialist compared with those who were aged 20–29 years.

#### Marital status

In Australia, respondents who had never been married were about 55% more likely to state that appointment making delays was not a difficult factor than those who were married [OR=0.42, CI=0.27–0.70, p<0.01].

#### Work status

In Thailand, respondents who worked full-time were about 55% more likely to state that appointment delay was not a difficulty factor the last time they required to see a doctor/specialist than those who worked part-time [OR=0.45, CI=0.24–0.84, p<0.05]. Those who were full-time workers were about 65% more likely to state that appointment delay was not a difficulty factor than those who worked for their family [OR=0.34, CI=0.23–0.49, p<0.001].

### Waiting time

Table 6 provides statistical results from binomial logistic regression models computed for the dependent variable waiting time.

**Table 6:** Six binomial logistic regression models for dependent variable: waiting time

**Country (Models for DV3)**	**1. AU**	**2. HK**	**3. JP**	**4. KR**	**5. TW**	**6. TL**
Model fit χ^2^ (df)	110.93 (21)***	108.49(5)***	94.06(10)***	14.49(4)**	21.20(5)**	48.49(14)***
N response/N total	814/1044 (78%)	666/674 (98.8%)	994/1000 (99.4%)	986/1006 (98%)	1082/1200 (90.2%)	1178/1200 (98.2%)
Nagelkerke R^2^	0.17	0.20	0.12	0.02	0.03	0.05
*Independent variable*	Waldχ^2^(df) p*	Waldχ^2^(df) p*	Waldχ^2^(df) p*	Waldχ^2^ df) p*	Waldχ^2^ (df) p*	Waldχ^2^(df) p*
	OR (95%CI)	OR (95%CI)	OR (95%CI)	OR (95%CI)	OR (95%CI)	OR (95%CI)
	Coefficient	Coefficient	Coefficient	Coefficient	Coefficient	Coefficient
**Sex**	-	’	’	’	’	’
Male (ref)						
Female	8.11χ^2^(1)**	’	’	’	’	’
	0.67 (0.51–0.88)					
	−0.54					
**Age**	’	’	20.63χ^2^(5)**	’	’	’
< 20 (ref)			1.00			
			0			
20–29	’	’	’	’	’	’
30–39	’	’	5.27χ^2^(1)*	’	’	’
			0.56 (0.34–0.92)			
			−0.58			
40–49	’	’	’	’	’	’
50–59	’	’	’	’	’	’
60+	’	’	’	’	’	’
**Work status**	40.22χ^2^(6)**	68.04χ^2^(5)***	’	’	’	14.01χ^2^(6)*
Full-time	1.00	1.00				1.00
(ref)	0	0				0
Part-time	4.82χ^2^(1)*	’	’	’	’	’
	0.62 (0.41–0.95)					
	−0.47					
Self-employed	’	’	’	’	’	’
Working for the family	’	’	’	’	’	11.16χ^2^(1)**
						0.52 (0.35–0.76)
						−0.66
Retired/pensioner	20.72χ^2^(1)***	66.34χ^2^(1)***	’	’	’	’
	2.45 (1.67–3.61)	0.68 (0.34–0.13)				
	0.90	−2.69				
Housewife/househusband	’	’	’	’	’	’
Student/	4.89χ^2^(1)*	***’***	’	’	’	’
Unemployed/	1.66 (1.06–2.60)					
Other	0.51					
**Subjective health**	28.51χ^2^(2)***	***’***	’	***Dissatisfied (ref)***	***Dissatisfied (ref)***	’
**satisfaction**	1.00			14.41χ^2^(2)**	12.68χ^2^(2)**	
Unhappy	0			1.00	1.00	
				0	0	
Fair	’	’	’	’	’	’
Happy	13.20χ^2^(1)***	’	’	***Satisfied***	***Satisfied***	’
	2.57 (1.54–4.27)			6.79χ^2^(1)**	5.02χ^2^(1)*	
	0.94			1.78 (1.15–2.74)	1.56 (1.06–2.29)	
				0.70	0.44	
**Financial situation in last**	15.85χ^2^(3)**	’	’	’	8.39χ^2^(3)*	14.74χ^2^(3)**
**year**	1.00				1.00	1.00
Save money (ref)	0				0	0
Just get by	5.98χ^2^(1)*	’	’	’	’	7.65χ^2^(1)**
	0.66 (0.45–0.91)					0.66 (0.49–0.89)
	−0.45					−0.41
Spent some savings	14.32χ^2^(1)***	’	’	’	7.26χ^2^(1)**	’
	0.38 (0.28–0.67)				0.55 (0.36–0.85)	
	−0.85				−0.59	
Spent savings & borrowed	3.72χ^2^(1)*	’	’	’	’	13.17χ^2^(1)***
money	0.56 (0.31–1.01)					0.48 (0.32–0.71)
	−0.58					−0.74
**Self-rated health**	’	’	44.73χ^2^(4)***	’	’	’
Very good (ref)			1.00			
			0			
Good	’	’	8.95χ^2^(1)**	’	’	’
			0.72 (0.58–0.89)			
			−0.33			
air	’	’	31.63χ^2^(1)***	’	’	’
			0.53 (0.43–0.66)			
			−0.63			
Bad	’	’	27.82χ^2^(1)***	’	’	’
			0.43 (0.31–0.59)			
			−0.85			
Very bad	’	’	8.34χ^2^(1)**	’	’	’
			0.41 (0.23–0.75)			
			−0.88			

ref: reference level, * p<.05; ** p<.01, *** p<.001.

#### Subjective health satisfaction

Australian respondents who stated they felt happy about their health were about 3 times more likely [OR=2.57, CI=1.54–4.28, p<0.001] to state that waiting times are not a difficulty factor.

For Korea, respondents who were satisfied with their health were approximately 1.8 times more likely [OR=1.78, CI=1.15–2.74, p<0.01] to indicate that waiting time was not a difficulty factor than respondents who were dissatisfied with their health.

Taiwan respondents who were satisfied with their health were over 1.5 times more likely [OR=1.56, CI=1.06–2.29, p<0.05] to state that waiting time was not a difficulty factor than those who were dissatisfied with their health.

#### Financial situation in last year

In Australia, those who stated they had saved money were about 35% more likely to state that waiting time was not a difficulty compared with those who ‘just got by’ [OR=0.66, CI=0.45–0.91, p<0.05]. Similarly, those who had saved money were about 60% more likely than those who had ‘spent some savings’ [OR=0.38, CI=0.28–0.67, p<0.05] and 45% more likely those who ‘spent savings and borrowed money’ [OR=0.56, CI=0.31–1.01] to state that waiting time was not a difficulty.

Taiwan respondents who had saved money were about 45% more likely than those who had spent some savings to state that waiting time was not a difficulty factor [OR=0.55, CI=0.36–0.85, p<0.01].

In Thailand, individuals who had saved money were about 35% more likely to state that waiting time was not a difficulty factor compared with those how ‘just got by’ [OR=0.66, CI=0.49–0.89, p<0.01]. Similarly, individuals who had saved money were nearly 50% more likely than respondents who spent savings and borrowed money to state that waiting time was not a difficulty factor [OR=0.48, CI=0.32–0.71, p<0.001].

#### Self-rated health

In Japan, those who rated their health as very good were about 30% more likely than respondents who rated their health as good to indicate waiting time was not a difficulty factor [OR=0.72,CI=0.58–0.89, p<0.05]. Again, those who rated their health as very good were about 50% more likely to indicate waiting time was not a difficulty factor than those who rated their health as fair [OR=0.53, CI=0.43–0.66, p<0.001] and approximately 60% more likely to indicate waiting time was not a difficulty factor than those who rated their health as bad [OR=0.43, CI=0.31–0.59, p<0.001] or very bad [OR=0.41, CI=0.23–0.75, p<0.01].

#### Sex

Only for Australia did the final logistic regression model show sex to be a significant indicator of waiting time difficulties, with males about 35% more likely than Australian females [OR=0.67, CI=0.51–0.88, p<0.01] to state that waiting time was not a difficulty factor on the last occasion they required to see a doctor/specialist.

#### Age

In Japan, than those who indicated to be under 20 years of age were about 40% more likely to state that waiting time was a not difficulty factor on the last occasion they required to see a doctor/specialist than those 30–39 years of age [OR=0.56, CI=0.34–0.92, p<0.05].

#### Work status

Respondents who worked full-time were about 40% more likely to state that waiting time was not a difficulty factor than those who worked part-time [OR=0.62, CI=0.41–0.95, p<0.05]. Respondents who were retired or pensioners were about 2.5 times more likely [OR=2.45, CI=1.67–3.61, p<0.001] to state that waiting time was not a difficulty factor, and respondents who were students/unemployed/other were about 1.5 times [OR=1.66, CI=1.06–2.60, p<0.05] more likely to state waiting time was a not difficulty factor compared with those who worked full-time.

In Hong Kong, respondents who worked full-time were about 30% more likely to state that waiting time was not a difficulty factor on the last occasion they required to see a doctor/specialist than those who stated they were retired/pensioners [OR=0.68, CI=0.34–0.13, p<0.001].

In Thailand, respondents who stated they worked full-time were about 50% more likely to state that waiting time was not a difficulty factor than those who worked for their family [OR=0.52, CI=0.35–0.76, p<0.01].

### Cost

Table 7 provides statistical results from binomial logistic regression models computed for the dependent variable cost.

**Table 7:** Six binomial logistic regression models for dependent variable: cost

**Country**	**1. AU**	**2. HK**	**3. JP**	**4. KR**	**5. TW**	**6. TL**
**(Models for DV4)**						
Model fit χ^2^ (df)	106.91 (20)***	121.19(13)***	99.37(13)***	102.49(15)***	76.75(9)***	29.28(8)***
N response/N total	912/1044 (87.4%)	665/674 (98.8%)	990/1000 (99%)	961/1006 (95.5%)	1081/1200 (90.1%)	1185/1200 (98.8%)
Nagelkerke R^2^	0.16	0.23	0.13	0.14	0.12	0.04
*Independent variable*	*Waldχ*^*2*^(df) p* OR	*Waldχ*^*2*^(df) p* OR	*Waldχ*^*2*^(df) p*	*Waldχ*^*2*^df) p*	*Wald*χ^*2*^(df) p*	*Wald*χ^*2*^(df) p*
	*(95%CI)*	*(95%CI)*	*OR (95%CI)*	*OR (95%CI)*	*OR (95%CI)*	*OR (95%CI)*
	*Coefficient*	*Coefficient*	*Coefficient*	*Coefficient*	*Coefficient*	*Coefficient*
**Sex**	-	’	’		’	’
Male (ref)	1.00			1.00		
	0			0		
Female	12.82χ^2^(1)***	’	’	4.58χ^2^(1)*	’	’
	0.54 (0.38–0.76)			0.72 (0.54–0.97)		
	−0.62			−0.33		
**Age**	’	’	(20–29 ref)	’	’	15.90χ^2^(5)**
< 20 (ref)						1.00
						0
20–29	’	’	16.72χ^2^(4)**	’	’	’
			1.00			
			0			
30–39	’	’	’	’	’	’
40–49	’	’	’	’	’	’
50–59	’	’	’	’	’	’
60+	’	’	8.08χ^2^(1)**	’	’	’
			1.89 (1.22–2.94)			
			0.64			
**Marital status**	15.51(5)**	32.12(4)***	’	13.09χ^2^(4)**	’	’
Never married (ref)	1.00	1.00		1.00		
	0	0		0		
Married	9.18(1)**	’	’	8.08χ^2^(1)**	’	’
	0.48 (0.30–0.77)			0.58 (0.40–0.85)		
	−0.73			−0.54		
Divorced	’	’	’	’	’	’
Widowed	’	20.12(1)***	’	7.02χ^2^(1)**	’	’
		19.81 (5.37–		0.38 (0.19–0.78)		
		73.05)		−0.96		
		2.97				
Separated	’	’	’	’	’	’
Cohabiting	9.23χ^2^(1)**	’	’	’	’	’
	0.38 (0.20–0.71)					
	−0.98					
**Household monthly**	9.72χ^2^(3)*	’	’	’	’	’
**adjusted income quartile**	1.00					
4^th^: quartile (ref)	0					
1^st^ quartile	’	’	’	’	’	’
2^nd^ quartile	8.11χ^2^(1)**	’	’	’	’	’
	0.52 (0.33–0.81)					
	−0.66					
3^rd^ quartile	’	’	’	’	’	’
**Work status**	21.17χ^2^(6)**	58.00χ^2^(5)***	’	’	21.23χ^2^(6)**	’
Full-time	1.00	1.00			1.00	
(ref)	0	0			0	
Part-time	’	’	’	’	18.30χ^2^(1)***	’
					0.31 (0.18–0.53)	
					−1.18	
Self-employed	’	0.00χ^2^(1)	’	’	’	’
		- (not given)				
		20.39***				
Working for the family	’	’	’	’	’	’
Retired/pensioner	15.92χ^2^(1)***	49–38χ^2^(1)***	’	’	’	’
	3.32 (1.84–5.99)	0.12 (0.06–0.21)				
	1.20	−2.15				
Housewife/househusband	3.90χ^2^(1)*	’	’	’	’	’
	2.03 (1.01–4.08)					
	0.71					
Student/	’	’	’	’	’	’
Unemployed/						
Other						
**Subjective health**	6.28χ^2^(2)*	***Dissatisfied (ref)***	’	***Dissatisfied***	’	’
**satisfaction**	1.00	10.65χ^2^(2)**		***(ref)***		
Unhappy	0	1.00		19.55χ^2^(2)***		
		0		1.00		
				0		
Fair	’	***Average***	’	’	’	’
		5.95χ^2^(1)*				
		2.41 (1.20–5.27)				
		0.92				
Happy	5.99χ^2^(1)*	***Satisfied***	’	***Satisfied***	’	’
	2.01 (1.15–3.50)	10.32χ^2^(1)**		16.00χ^2^(1)***		
	−0.70	3.24(1.58–6.62)		2.68 (1.65–4.33)		
		1.17		0.98		
**Financial situation in last**	18.19χ^2^(3)***	’	18.44χ^2^(3)***	12.54χ^2^(3)**	41.39χ(3)***	13.40χ^2^(3)**
**year**	1.00		1.00	1.00	1.00	1.00
Save money (ref)	0		0	0	0	0
Just get by	2.01χ^2^(1)*	’	’	’	24.47χ^2^(1)***	9.98χ^2^(10**)
	0.67 (0.45–0.99)				0.32 (0.21–0.51)	0.55 (0.38–0.80)
	−0.40				−1.13	−0.61
Spent some savings	’	’	11.76χ^2^(1)**	’	22.44χ^2^(1)***	7.46χ^2^(1)**
			0.53 (0.37–0.76)		0.25 (0.14–0.44)	0.45 (0.25–0.80)
			−0.64		−1.41	−0.80
Spent savings & borrowed	17.73χ^2^(1)***	’	12.50χ^2^(1)***	12.13χ^2^(1)***	33.38χ^2^(1)***	9.22χ^2^(1)**
money	0.31 (0.18–0.4)		0.38 (0.23–0.65)	0.32 (0.17–0.61)	0.16 (0.09–0.30)	0.48 (0.30–0.77)
	−1.17		−0.96	−1.13	−1.83	
**Self-rated health**	’	’	41.14χ^2^(4)***	’	’	’
Very good (ref)			1.00			
			0			
Good	’	’	’	’	’	’
Fair	’	’	8.04χ^2^(1)**	’	’	’
			0.48 (0.29–0.80)			
			−0.74			
Bad	’	’	14.63χ^2^(1)***	’	’	’
			0.27 (0.14–0.53)			
			−1.31			
Very bad			2.44χ^2^(1)			
			0.37 (0.11–1.29)			
			−1.00			
**Whether respondent has**	’	’	’	1.00	’	’
**chronic health condition**				0		
Yes (ref)						
No	’	’	’	χ2(1)=6.70*	’	’
				2.51 (1.25-5.04)		

Ref: reference level, * p<.05; ** p<.01, *** p<.001.

* Levels too low in one item (widowed f=3; separated f=1).

** Levels too low in one item (separated f=4).

*** Levels too low in one item (self-employed f=7).

#### Household monthly/annual income

In Australia those who were in the highest income quartile were approximately 50% more likely to state cost was not a difficulty factor than respondents who belonged to the second lowest income quartile [OR=0.52, CI=0.33–0.81, p<0.01].

#### Subjective health satisfaction

Australian respondents who indicated they were happy with their health were twice as likely [OR=2.01, CI=1.15–3.50, p<0.05] to state that cost was not a difficulty factor than those who were unhappy with their health.

In Hong Kong, individuals who were neither satisfied nor dissatisfied with their health were about 2.5 times more likely [OR=2.41, CI=1.20–5.27, p<0.05] to indicate that cost was not a difficulty factor than those who were dissatisfied with their health. Respondents who were satisfied with their health were about 3.2 times more likely [OR=3.24, CI=1.58–6.62, p<0.01] to state cost was not a difficulty factor than those who were dissatisfied with their health.

Korean respondents who were satisfied with their health were about 2.7 times more likely [OR=2.68, 1.65–4.33, p<0.001] to state that cost was not a difficulty factor than those who were dissatisfied with their health.

#### Financial situation in last year

Australians who had saved money were about 60% more likely than those who ‘just got by’ [OR=0.67, CI=0.45–0.99, p<0.05] and about 70% more than those who had spent savings and borrowed money [OR=0.31, CI=0.18–0.40, p<0.001] to state that cost was not a difficulty factor.

In Japan, those who had saved money in the last year were about 40–50% more likely to state that cost was not a difficulty factor compared with respondents who stated that in the last year they spent some savings [OR=0.53,CI=0.37–0.76, p<0.01] or spent savings and borrowed money [OR=0.38, CI=0.23–0.65, p<0.001].

As for Korean respondents, those who had saved money were about 70% more likely than those who had spent savings and borrowed money to state that cost was not a difficulty factor [OR=0.32, CI=0.17–0.61, p<0.001].

Taiwanese respondents who stated they had saved money in the last year were about 70% or 75% respectively more likely to state that cost was not a difficulty factor than those who stated ‘just got by’ [OR=0.32, CI=0.21–0.51, p<0.001], or ‘spent some savings’ [OR=0.25, CI=0.14–0.44, p<0.001]. Those who had saved money were about 85% more likely to indicate that cost was not a difficulty factor compared with those who had spent savings and borrowed money [OR=0.16, CI=0.09–0.30, p<0.001].

In Thailand, respondents who had saved money in the last year were about 50% or 55% respectively more likely to state that cost was not a difficulty factor than those who stated ‘just got by’ [OR=0.55, CI=0.38–0.80, p<0.01], or ‘spent some savings’ [OR=0.45, CI=0.25–0.80, p<0.01]. Similarly, those who had saved money were about 55% more likely those who had spent savings and borrowed money to indicate that cost was not a difficulty factor [OR=0.48, CI=0.30–0.77, p<0.01].

#### Self-rated health

In Japan, those who rated their health as very good were about 50% more likely to indicate cost was not a difficulty factor compared to respondents who rated their health condition as fair [OR=0.48, CI=0.29–0.80, p<0.01]. Those who rated their health as very good were nearly 75% more likely to indicate that cost was not a difficulty factor than those who rated their health as bad [OR=0.27, CI=0.14–0.53, p<0.001].

#### Chronic health condition

In Korea, respondents who indicated having no chronic health condition were about 2.5 times more likely [OR=2.51, CI=1.25–5.04, p<0.05] to state that cost was not a difficulty factor compared with those who suffered from a chronic health condition.

#### Sex

In Australia, males were about 45% more likely to state that cost was not a difficulty factor on the last occasion they required to see a doctor/specialist than females [OR=0.54, CI=0.38–0.76, p<0.001].

Males in Korea were about 30% more likely than females [OR=0.72, CI=0.54–0.97, p<0.01] to indicate that cost was a not difficulty factor on the last occasion they required to see a doctor/specialist.

#### Age

In Japan, respondents who were 60 years or older were nearly twice as likely [OR=1.89, CI=1.22–2.94, p<0.01] to state that cost was not a difficulty factor on the last occasion they required to see a doctor/specialist than those who were between 20 and 29 years old.

#### Marital status

In Australia, respondents who had never married were about 50% more likely to state that cost was not a difficulty factor than those who were married [OR=0.48, CI=0.30–0.77, p<0.01].

In Hong Kong, respondents who were widowed were about 19 times more likely [OR=19.81, CI=5.37–73.05, p<0.001] to state that cost difficulties were not an issue on the last occasion they required to see a doctor/specialist than those who had never married.

#### Work status

Australians who were retired/pensioners were about 3.3 times more likely [OR=3.32, CI=1.84–5.99, p<0.001] to state cost was not a difficulty on the last occasion they required to see a doctor/specialist than full-time workers. Those who were housewives or househusbands were twice as likely [OR=2.03, CI=1.00–4.08, p<0.05] to state cost was not a difficulty than those who were full-time workers.

In Hong Kong, respondents who worked full-time were approximately 90% more likely to state that cost was a not difficulty factor compared with those who were retired or pensioners [OR=0.12, CI=0.64–0.21, p<0.001].

In Taiwan, respondents who worked full-time were about 70% more likely to state that cost was not a difficulty factor on the last occasion they required to see a doctor/specialist than those who worked part-time [OR=0.31, CI=0.18–0.53, p<0.01].

## Discussion

The aim of this manuscript is to highlight disparities and differences in terms of the factors that seem to be important to distinguishing between poor and good access to healthcare across six countries. In the following we discuss these findings in relation to healthcare provision within each of these countries. We do not provide a comprehensive explanation of our findings but rather, we provide a level of interpretation around the country context for each of the findings. Our results for each country should be used as baseline data or as the basis for an intervention by researchers with knowledge of the local, social, cultural and political context.

### Australia

The findings identified that respondents who are unhappy with their health have difficulty accessing services due to distance. This is obviously worrying, and is an example of the ‘inverse care law’ [23]. This is of concern given the potential for increasing inequities in health outcomes.

The findings regarding females having difficulty accessing services is also concerning. Studies from both high- and low income countries show higher rates of morbidity among women than men [67]. However, in line with previous research, the findings may also provide further evidence to suggest that health services are under-utilised (thereby rendering difficulties in access irrelevant) by men which has obvious implications for men’s health in Australia. Data from the UK suggest that men are less likely than women to consult their general practitioners but have higher admission rates for chronic disease which suggests that men’s health policy efforts may need to focus on promotion and prevention [68].

Finances, inclusive of annual income and difficulty with finances in the past year, were also found to be a predictor of poor access to healthcare across distance, appointment, waiting time and cost. Previous research conducted in Australia identified that patients with low socioeconomic status are likely to have shorter consultations with GPs despite the fact that, as a group, they have a significantly higher need for care [69]. Although Australia has publically funded healthcare via Medicare [70], waiting times and difficulties in being granted a medical appointment continue to be a problem in Australia. Individuals have the option of purchasing private services via out of pocket payment or private insurance schemes which minimises waiting times and permits more timely access to specialists via private appointments. However, figures from the ABS 2007–08 National Health Survey indicate that only half (53%) of the Australian population aged 15 years and over have private health insurance [71].

### Hong Kong

Respondents in Hong Kong who spent their savings in the past year and who fell in the lowest income quartile were found to have difficulties accessing healthcare due to distance. We cannot explain this finding and suggest that more research is needed to understand the role of poverty in determining geographical inequity in access to services.

Older individuals (over 60, pensioners, retired) were found to have problems accessing health services do to difficulties getting appointments, waiting times and cost. At the time of data collection (the 2008–09 financial year), the Government of Hong Kong launched the Elderly Health Care Voucher Pilot Scheme (HCVS) for three years to provide five healthcare vouchers of $50 each to elders aged 70 or above annually to partially subsidise their use of private primary healthcare (PHC) services. There was recognition by government that programs were needed for the elderly to be granted better access to care and a continuity of care from their chosen providers. The additional aim was to reduce elderly reliance on public healthcare resources so that other members of the public who are in need of primary healthcare services would be benefit indirectly. Results from an interim review on the Scheme have demonstrated its success and the Government has decided to extend the Scheme for another three years from 1 January 2012 to 31 December 2014, increasing the voucher amount entitled by each eligible elder from $250 to $500 per year [72]. Our recommendation is to obtain a repeat measure of access to healthcare as a means of identifying the impact of this policy on access to healthcare for the elderly.

### Japan

Japan has been argued to be one of the most equitable one-tiered (solely private) health systems in the world [3,73]. Roughly 80% of Japan’s hospitals and 94% of its physician-run offices (referred to as clinics) are privately operated; however all care is provided under a nationally uniform fee schedule which means that neither insurers nor providers have the freedom to negotiate individual fee schedules [74,75]. Every citizen must be attached to one scheme but insurance premiums are calculated on the basis of income. Individuals can be on public assistance which is the only situation under which an individual does not have to be covered by an insurance scheme.

Our results identified that difficulties in the financial year prior to data collection was predictive of difficulties in accessing healthcare due to cost. Although insurance premiums are regulated, as noted above, these premiums are calculated based on income. It is possible that healthcare premiums are calculated in a manner that does not take into account current/recent income (i.e. people are charged more than they can afford until income is reassessed).

Additionally, in contrast to trends across the other countries, being satisfied with one’s health was a predictor of difficulties in gaining an appointment. We cannot explain this finding and suggest that more research is needed to understand if health care service access is prioritised according to need (i.e. people dissatisfied with their health are priority).

### Korea

Respondents who spent savings or borrowed money in the year prior to data collection were found to have difficulty in accessing care due to cost. This may be due to the nature of the South Korean healthcare system. All Koreans are entitled to the National Health Insurance Program (NHIP); however, Song (2009) reported that only 57.7% were enrolled in this service in 2006. Additionally, for those who do enrol, it is required that individuals pay a portion of the healthcare costs, which vary according to the level and type of healthcare institution being used. Some rates are as high as 50% of the total cost of treatment (i.e. for general hospital visits and treatments). Those who cannot afford co-payments are covered by the Medical Aid Program which pays for all medical expenses for those who are unable to pay. However, only 3.7% of South Koreans are eligible for this service (Song 2009).

### Taiwan

A National Health Insurance (NHI) scheme covers 99% of Taiwan’s population [76]. The objective of the NHI is to provide equal access to healthcare for all citizens and to maintain healthcare expenditure at manageable levels [77]. By law, regardless of age, gender, or employment status, any Taiwanese citizen with a local ID card or a foreign national living in Taiwan with an Alien Resident Certificate (ARC) is required to enrol in the program. Additionally, those eligible for the system must participate in the system (unless they lose their eligibility by being convicted of a crime, disappearing, giving up their Taiwan citizenship, moving abroad or having their Alien Resident Certificate expired) [78]. The premium for the insurance scheme is 100% subsidized for households below the poverty line. Despite this, we identified that difficulties in finances in the year prior to data collection and working part time were predictors of difficulties in access due to cost. Our findings may be explained by the fact that part-time workers or individuals who had difficulty in the financial year prior to data collection may not be eligible for subsidized premiums [79]. While the scheme is designed so that no single individual living in Taiwan will be denied healthcare for lack of means, low-to-middle income earners may fall short of eligibility criteria for access to these subsides.

### Thailand

Employment status was the main predictor for difficulty in accessing healthcare in Thailand. Individuals who work for their family, do part time work, work within the household or are unemployed were found to have difficulty accessing services due to distance, difficulties getting an appointment and/or waiting times. This may be explained in part by the structure of the Thai social health insurance program. Thai individuals working in the private sector are provided with health insurance under the Civil Servant Medical Benefit Scheme (CSMBS) or the Social Security Scheme. However, this social insurance scheme does not cover people working outside of the private sector, non-working spouses, child dependents or other family members. While there is a scheme used by the remainder of the population, referred to as the 30-Baht Scheme whereby a person pays 30 Baht to receive access to any health service required, Thailand still struggles to extend coverage to the majority of its population [80]. The 30-Baht Scheme may also explain the finding that respondents who had financial difficulties in the year prior to the data collection also found it difficult to access service due to cost, whereby some individuals may not be able to afford this prescribed payment.

Consistent across the findings, poor health and low income were identified as difficulties in access due to distance. In a similar vein, low income was found to be a predictor for difficulties in access due to cost. These findings echo previous cross-country research investigating access (Burkina Faso, Guatemala, Kazakhstan, Kyrgyzstan, Paraguay, South Africa, Thailand, Zambia), whereby it was found that wealthier population groups have a higher probability of obtaining healthcare when they need it, are more likely to be seen by a doctor, and to receive medicines [81]. Our findings present further evidence of the inverse care law - population groups with the highest levels of healthcare need (economically deprived, poor health status) often have the poorest access to services [23]. The findings suggest that regionally (Asia-Pacific), policy makers need to recognise the increasing inequities for these population groups and react accordingly which is inclusive of addressing the foundations of these inequities through policy/system change.

Excluding poor health and income, the picture is more complex when looking at trends in predictors within countries. For Thailand, the poorest level of access appears to be for respondents who work within the household whereas in Taiwan, part-time work is associated with difficulties in access. Within Hong Kong, results suggest that older (above 60) and retired individuals have the poorest access and within Australia, females and married individuals are the worst off. The countries involved in this study are in varying stages of socioeconomic and health development. The needs and functions of each country’s healthcare system therefore vary and require policy reform tailored to the contours of their health systems. However, some conclusions can be drawn from the successes of these countries. While we identified health access to be problematic for older individuals in Hong Kong, recent policy has shifted to increase access to services for older individuals which indirectly, has been suggested to be delivering more equitable service for the remainder of citizens. Likewise, Taiwan has developed a system which was specifically designed to provide universal services for all individuals. Our findings also identify the need for national policies to address country-specific inequities.

## Conclusion

It is recognised trans-nationally that public health policy and practice needs to focus on addressing the Social Determinants of Health in order to increase the health of the most vulnerable and disadvantaged groups [5]. This research has identified a number of social determinants which predict inequities in access to healthcare in six countries across Asia-Pacific. We have discussed our findings in relation to the political climate of healthcare in each of the six countries.

Recognition of these inequities, from a policy perspective, is essential for health sector policy decision-making [81]. Health policies shape health systems, and consequently, the broader determinants of health [82]. These policies should be informed by an understanding of the difficulties experienced by individuals when trying to obtain medical attention [36]. Despite the differences in political and economic climate in the countries under analysis, our findings highlight patterns of inequity which require policy responses. We argue that our data should be used as a means of deciding the most appropriate policy response for each country which includes, rather than excludes, socially marginalised population groups [83]. These findings should be of interest to those involved in health policy, but also in policy more generally because as we have identified, health is influenced by determinants outside of the health system [84].

Our results also provide baseline measures for identifying where and how policy should be altered to enable equitable access. Furthermore, as suggested for reforms in Hong Kong, these measures should be used for future policy evaluation to identify if shifts in policy have indeed improved access for the marginalised groups identified. An investigation into the possible causes of these inequities, and plans to address them, should be the focus of government agendas.

At this point, the authors wish to recognise and briefly discuss potential shortcomings stemming from aspects of the design, data collection and analytical procedures associated with this research. As presented in the results section, the amount of variance explained by the factors included in the models specified for all countries separately and combined was generally small as indicated by low Nagelkerke R2 estimates. It is therefore acknowledged that the (socio) demographic predictors included in the current investigation do not achieve substantial contributions to explaining response differences, hence further variables need to be investigated which might carry stronger predictive qualities for the outcome variables examined. Despite the comparatively small amount of variance explained, the models specified were (highly) significant which is taken as being indicative of small, yet significant contributions of (socio) demographic variables to various aspects of access to healthcare.

It is furthermore acknowledged that differences in survey format, the exact formulation of individual survey items and response options, as well as data collection in the different settings have potentially introduced biases when aggregating data and/or comparing the results. Maintaining data integrity and keeping the data collected as close to their raw form as possible meant particular predictor as well as outcome variable levels had to be collapsed and comparisons drawn between variables which are not 100% identical in terms of meaning, response format, etc. Several problems arise from this process, including the loss of fine-grained differences for certain predictor and outcome variable levels which may have resulted in slightly different make-up of significant regression models. Whilst this undoubtedly creates limitations for the extent to which the current findings can be generalised, this study presents the first attempts to conduct a secondary analysis of cross-country data collected for this specific purpose using study materials which have been generated with the aim to be applicable in different settings. The authors therefore acknowledge that limitations apply which mean the current results need to be interpreted and generalised with caution, and that replication is required to strengthen the existing body of evidence. Nevertheless, given the relative paucity of data in this area of interest, the current results add substantially to our current understanding of variables affecting access to healthcare in different countries.

## Endnotes

^1^The broader study from which the data in this manuscript emanate was based on Social Quality Theory (SQT). We have provided detailed manuscripts on SQT elsewhere Within Social Quality Theory, access to healthcare is identified as an indicator of social quality [52].

^2^Japan (Chiba University), Taiwan (National Taiwan University), Hong Kong (Chinese University Hong Kong), Thailand (King Prajadhipok Institute), Korea (Seoul National University).

^3^The Japanese sample was not stratified according to health insurance type which may have implications for the interpretation of the Japanese data.

## Competing interests

The authors declare that they have no competing interests.

## Authors’ contributions

CNL and SBM participated in the design of the study, piloting and data collection. CNL and LM carried out the data analysis. All authors drafted the manuscript. PRW wrote the grant application and received funding for the study. All authors read and approved the final manuscript.

## Pre-publication history

The pre-publication history for this paper can be accessed here:

http://www.biomedcentral.com/1472-6963/13/238/prepub
